# Estimated prevalence of potentially damaging variants in the leptin gene

**DOI:** 10.1186/s40348-017-0074-x

**Published:** 2017-11-03

**Authors:** Adriana Nunziata, Guntram Borck, Jan-Bernd Funcke, Katja Kohlsdorf, Stephanie Brandt, Anke Hinney, Barbara Moepps, Peter Gierschik, Klaus-Michael Debatin, Pamela Fischer-Posovszky, Martin Wabitsch

**Affiliations:** 1 Division of Pediatric Endocrinology and Diabetes, Department of Pediatrics and Adolescent Medicine, University Medical Center Ulm, Eythstr. 24, D-89075 Ulm, Germany; 2 Institute of Human Genetics, University of Ulm, Albert-Einstein-Allee 11, D-89081 Ulm, Germany; 3 Department of Child and Adolescent Psychiatry, Universität Duisburg-Essen, University Hospital Essen, Virchowstr. 174, D-45147 Essen, Germany; 4 Institute of Pharmacology and Toxicology, Universität Ulm, Albert-Einstein-Allee 11, D-89081 Ulm, Germany

**Keywords:** Monogenic obesity, Leptin deficiency, ExAC, Exome sequencing, Obesity prevalence

## Abstract

**Background:**

Mutations in the leptin gene (LEP) can alter the secretion or interaction of leptin with its receptor, leading to extreme early-onset obesity. The purpose of this work was to estimate the prevalence of heterozygous and homozygous mutations in the leptin gene with the help of the Exome Aggregation Consortium (ExAC) database (http://exac.broadinstitute.org/about).

**Results:**

The ExAC database encompasses exome sequencing data from 60,706 individuals. We searched for listed leptin variants and identified 36 missense, 1 in-frame deletion, and 3 loss-of-function variants. The functional relevance of these variants was assessed by the in silico prediction tools PolyPhen-2, Sorting Intolerant from Tolerant (SIFT), and Loss-Of-Function Transcript Effect Estimator (LOFTEE). PolyPhen-2 predicted 7 of the missense variants to be probably damaging and 10 to be possibly damaging. SIFT predicted 7 of the missense variants to be deleterious. Three loss-of-function variants were predicted by LOFTEE. Excluding double counts, we can summarize 21 variants as potentially damaging. Considering the allele count, we identified 31 heterozygous but no homozygous subjects with at least probably damaging variants. In the ExAC population, the estimated prevalence of heterozygous carriers of these potentially damaging variants was 1:2000. The probability of homozygosity was 1:15,000,000.

We furthermore tried to assess the functionality of ExAC-listed leptin variants by applying a knowledge-driven approach. By this approach, additional 6 of the ExAC-listed variants were considered potentially damaging, increasing the number of heterozygous subjects to 58, the prevalence of heterozygosity to 1:1050, and the probability of homozygosity to 1:4,400,000.

**Conclusion:**

Using exome sequencing data from ExAC, in silico prediction tools and by applying a knowledge-driven approach, we identified 27 probably damaging variants in the leptin gene of 58 heterozygous subjects. With this information, we estimate the prevalence for heterozygosity at 1:1050 corresponding to an prevalence of homozygosity of 1:4,400,000 in this large pluriethnic cohort.

**Electronic supplementary material:**

The online version of this article (10.1186/s40348-017-0074-x) contains supplementary material, which is available to authorized users.

## Background

The brain is the central regulator of body weight by balancing energy intake and expenditure. Both genetic and environmental factors affect this balance with underweight and obesity as extreme outcomes of its disturbance.

The rapid development of faster and cheaper sequencing methods enables the identification of rare monogenic disease including variants causing disturbances in weight regulation via impaired leptin-melanocortin signaling [1]. Some important monogenic forms are caused by mutations in, e.g., leptin (LEP), the leptin receptor (LEPR), pro-opiomelanocortin (POMC), the signaling component Src homology 2B adapter protein 1 (SH2B1), or the melanocortin-4 receptor (MC4R) (see Additional file 1: Table S1 for details). The estimated prevalence of mutations causing monogenic forms of obesity ranges from 1 to 5% in severely obese subjects, with mutations in MC4R being most common [2].

**Fig. 1:**
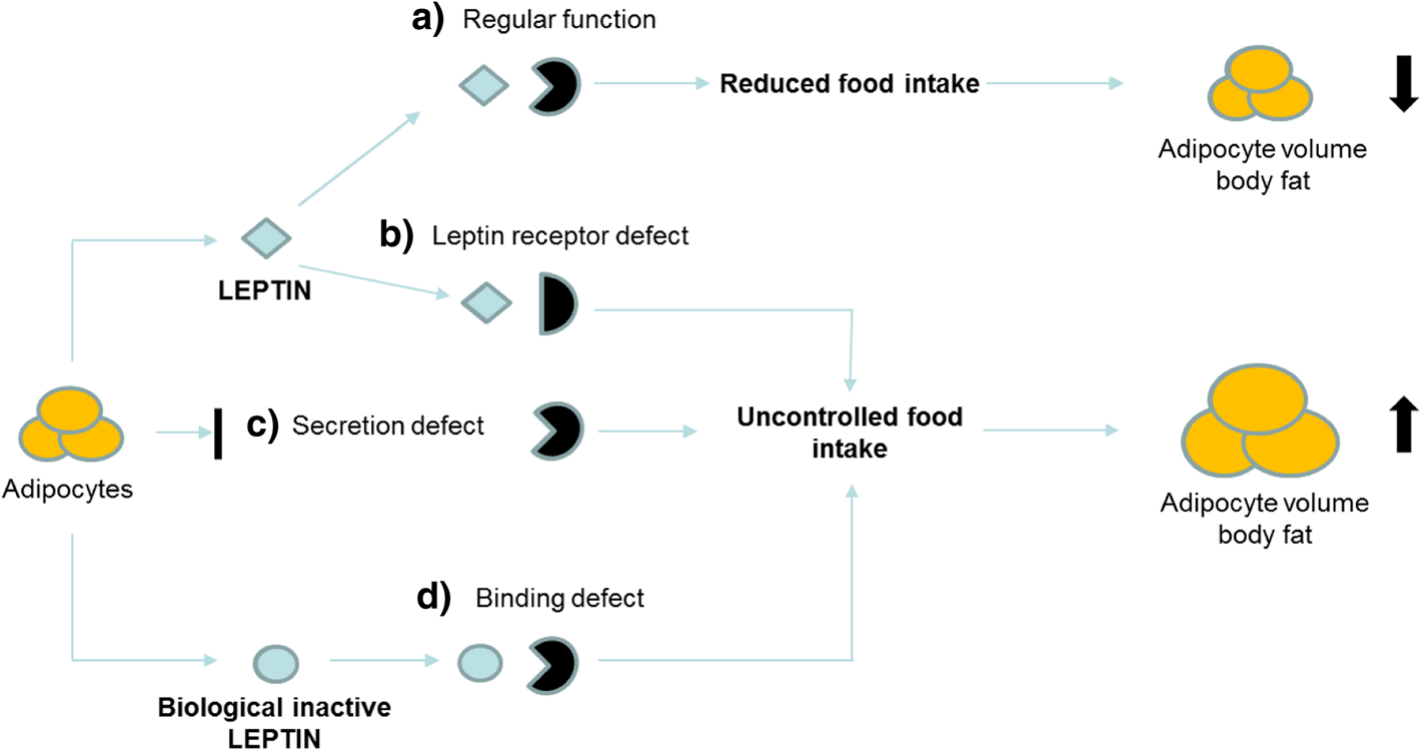
Pathologies in the leptin and leptin receptor pathway*.* Simplified representation of the secretion of leptin in the adipocytes and the interaction with the leptin receptor in the hypothalamus. **a** Regular function of leptin. Following secretion from adipocytes, leptin can activate the hypothalamic leptin receptor which reduces food intake and results in decreases in adipocyte volume and body fat. **b**–**d** Functional defects of leptin or the leptin receptor result in uncontrolled food intake and increases in adipocyte volume and body fat. Mutations can either affect the leptin receptor (**b**), the production and secretion of leptin (**c**), or the interaction of leptin with its receptor (**d**)

The exact prevalence of variants in the gene-encoding leptin (LEP) is not known. In contrast to, e.g., MC4R variants, pathological leptin deficiency occurs only in subjects with biallelic mutations. In 1997, Montague et al. were the first to describe two subjects with a homozygous mutation in the leptin gene. This frameshift mutation, p.Gly133Valfs*15, is deemed to be underproduced due to nonsense-mediated mRNA decay and not secreted due to aberrant cellular transport [3] (see Fig. 1c). In the affected subjects, the disturbance of the leptin-melanocortin pathway led to extreme early-onset obesity as well as metabolic disorders, hypogonadotropic hypogonadism, and suppressed immune function [3–6]. In 2015, we described the existence of biologically inactive leptin due to missense mutations (p.Asp100Tyr and p.Asn103Lys, see Fig. 1d) [7, 8]. Up to now, 53 subjects affected by leptin gene variants with either proven or suggested functional disturbance have been described in the literature (see Additional file 1: Table S2) [1–19, 21, 22, 47]. It should be noted that there might be several cases which are either not diagnosed or not published hampering the estimation of prevalence via literature analysis. Identifying monogenic forms of obesity is a psychological relief for the patient and relatives, sometimes enabling treatment with, e.g., metreleptin or setmelanotide [7, 10, 23–25]. In our outpatient clinic, we encounter almost daily patients struggling with extreme obesity, and the pediatricians face the question if cost-intensive sequencing is required. With this work, we aim to estimate the prevalence of heterozygous and homozygous mutations in the leptin gene with the help of the Exome Aggregation Consortium (ExAC) database. Taking congenital leptin deficiency as our example, we moreover want to outline this database as a helpful tool to estimate the prevalence of rare monogenetic diseases [26]. Conversely, we want to analyze the allele frequency of published mutations in the leptin gene, known to cause severe obesity when inherited homozygously.

## Methods

### ExAC

The ExAC database (http://ExAC.broadinstitute.org/; Accessed: 25 April 2017) collects exome sequencing data from worldwide studies including data from, e.g., the 1000 Genomes Project. Further information on participating studies and ethnic distribution are available in the FAQ of the ExAC database (http://ExAC.broadinstitute.org/faq). After exclusion of, e.g., low quality data, related individuals, and individuals with severe pediatric disease, data was available from 60,706 individuals with a lack of data from Middle Eastern and Central Asian populations. All data is based on the human genome assembly GRCh37/hg19. The mean coverage for the leptin gene was ~ 80× [26].

### Variants in the LEP and their functional analysis

We searched the gene of interest (Leptin/LEP, canonical transcript ENST00000308868) and focused on missense and loss-of-function (LoF) variants.

ExAC provides more detailed information on each variant such as ethnical distribution and allele frequency. Using in silico models, namely PolyPhen-2 (Polymorphism Phenotyping v2), SIFT (Sorting Intolerant From Tolerant), and LOFTEE (Loss-Of-Function Transcript Effect Estimator), ExAC estimates the impact of a given variant on protein function.

PolyPhen-2 categorizes the variants in three groups: benign, possibly damaging, and probably damaging. SIFT categorizes in two groups: tolerated and deleterious. Single nucleotide changes in splice acceptor/splice donor side and nonsense variants were labeled as LoF and analyzed with the help of LOFTEE (see Additional file 1: Table S3).

In addition, we estimated the function of leptin variants by applying a knowledge-driven approach, based on findings described in mutagenesis studies. Leptin has three binding sides (BS). The role of BS I is yet undefined but is unlikely to be involved in interactions with the functional leptin receptor isoform. BS II is needed for receptor binding, whereas BS III is required for signaling [27–29]. Variants located close to those binding sites (accepted distance ≤ 4 amino acids) or nearby the disulfide bond in position of cysteines 117 and 167 of the immature protein required for structural stability were classified as potentially functionally damaging [28, 30–32]. We did not distinguish between different chemical properties of the amino acids. In addition, it cannot be excluded that variants in other areas may alter the transcription and therefore the function of the protein.

Based on these findings, we calculated the probability of hetero- and homozygosity with the binominal distribution predicted by the Hardy-Weinberg principle assuming a perfect population. *p*2 + 2*pq* + *q*2 = 1 (*p* = allele frequency of allele A; *q* = allele frequency of allele B).

We compiled a list of subjects homozygous for leptin variants and severe obesity based on literature research (PubMed, OMIM). With the help of the ExAC browser, we looked for the allele frequency of these variants [1–18, 21, 22, 47].

## Results

The functional relevance of variants in the leptin gene listed in ExAC was assessed by the in silico prediction tools PolyPhen-2, SIFT, and LOFTEE. PolyPhen-2 predicted 10 of the missense variants to be possibly damaging and 7 to be probably damaging. SIFT predicted seven of the missense variants to be deleterious. Three LoF variants were predicted by LOFTEE (see Additional file 1: Table S3).

Our own analysis revealed 6 additional variants to be located closer than ≤ 4 amino acids to BS II, BS III, or the disulfide bond (p. Ile35del, p.Lys36Arg, p.Ile45Val; p.Ile63Leu, p.Ala137Ser, p.Gly166Arg) and therefore potentially damaging. None of those 6 variants was predicted as functionally disturbing by in silico models used in ExAC.

Based solely on PolyPhen-2, SIFT, and LOFTEE analyses (excluding double counts), we can summarize 21 variants as potentially damaging. Considering the allele count, we identified 31 heterozygous but no homozygous subjects with variants predicted to be at least probably damaging in ExAC. With this, the estimated prevalence of heterozygosity was ≈1:2000 and the corresponding probability of homozygosity was ≈1:15,000,000 in the ExAC population.

When we included the 6 variants deemed to be potentially damaging by our own functional assessment, we identified 58 heterozygous subjects increasing the prevalence of heterozygosity to ≈1:1050 and the corresponding probability of homozygosity of ≈1: 4,400,000.

Summarizing the data from the literature of homozygous severely obese subjects with a homozygous LEP mutation, we identified 53 subjects with 12 distinct variants in the leptin gene. Of the identified variants, 6 were missense, 1 was an in-frame deletion, and 5 were LoF variants (see Additional file 1: Table S2). Only three of those variants are listed in ExAC database (p.Gly133Valfs*15, p.Asn103Lys and p.Ile35del; see Table 1) and described as functionally damaging. From the 12 variants reported in the literature, p.G133Vfs*15 is the most frequent with 30 affected subjects, all of Pakistani origin, suggesting that it constitutes a founder mutation. In accordance with this, p.G133Vfs*15 displayed the highest allele count (of mutations described in the literature) localized exclusively in South Asian population in ExAC.

**Table 1:** Variants in the leptin gene listed in the ExAC database and reported in the literature to cause congenital leptin deficiency in the homozygous state

Variant	Allele number	Allele frequency	Allele count	Homozygous	Population
p.Gly133Valfs*15	120,412	0.00003322	4	0	South Asian
p.Asn103Lys	121,400	0.00001647	2	0	European (non-Finnish)
p.Ile35del	120,412	0.00001647	2	0	South Asian & European (non-Finnish)

*indicates a stop codon

## Discussion

The ExAC database is a tool with which one can predict if a gene variant is a polymorphism or a functionally damaging variant that may be a disease-causing mutation. Exemplifying this, roughly 200 variants that were previously believed to be disease-causing mutations were now revealed to be mostly harmless polymorphisms by use of the database [26, 34]. In 2017, ExAC aims to add 120,000 exome and 20,000 whole genome sequences [26]. ExAC is a helpful tool to avoid cost-/time-intensive screenings and misinterpretations of detected variants. Nevertheless, ExAC has some limitations. Studies, which provided the data, screened mostly cohorts with specific underlying disease, e.g., inflammatory bowel disease, type 2 diabetes, or schizophrenia (further information about the used cohorts can be looked up in the FAQ section of the website http://exac.broadinstitute.org/faq), and thus represent a selected population. Subjects with severe pediatric disease were excluded. This inevitably leads to exclusion of some variants causing severe autosomal dominant disease. ExAC used cohorts throughout the world. Still, there is a lack of information from the Middle Eastern and Central Asian population. This data would especially be interesting for the leptin gene considering that most described cases with monogenic leptin deficiency are from Middle Eastern origin [3, 9, 10, 13, 14, 17, 18].

It should also be noted that ExAC does not include variants with larger deletions or duplications due to technical reasons. At this point of time, ExAC does not provide information about phenotypic characteristics such as BMI for ethical reasons. Finally, the validity of PolyPhen-2/SIFT/LOFTEE is based on in silico predictions and is thus somehow limited and can hardly replace functional in vitro studies [3, 6, 7]. The p.Ile35del variant for instance, was not predicted as functionally damaging; however, it is described in Fatima et al. and Saeed et al. to provoke obesity in homozygous conditions [13, 16]. We note that other algorithms for predicting the effects of missense variants exist (see, e.g., the programs referenced on https://omictools.com/functional-predictions-category) as well as another (larger) exome and genome sequence database, gnoMAD (http://gnomad.broadinstitute.org/).

With regard to the leptin gene, we note that pathological leptin deficiency occurs only in subjects with biallelic mutations and is therefore much less frequent than, e.g., MC4R mutation (which can lead also in the heterozygous status to disease manifestation). There is no evidence for a pathogenic phenotype in subjects with heterozygous LEP mutations. We also note that our estimation does not account for compound heterozygous LEP mutations that may cause severe obesity.

We conclude that the estimated prevalence for homozygous leptin variants is very low at one case in 15 million (ExAC) or 4.4 million (including our analysis). Practically this should demonstrate that the leptin gene is not a candidate for routine genetic screening in obese patients, but it will probably be included in multigene panels for monogenic obesity. This holds true especially as leptin deficiency, although rare, has therapeutic consequences. We therefore consider more refined clinical criteria for preliminary investigation before time- and cost-intensive genetic screening including the survey of weight development in early childhood and a recently developed assay enabling the measurement of bio-active leptin (see Fig. 1d) [35].

## Conclusion

ExAC is a unique collection of data comprising a high variety of ethnicities. It constitutes a free resource that simplifies the access to exome sequence data remarkably. It is a user-friendly tool which a clinician in cooperation with a genetic laboratory can easily apply in his daily routine to estimate the prevalence of variants. Using exome sequencing data from ExAC, in silico prediction tools and a knowledge-driven approach, we identified 27 probably damaging variants in the leptin gene of 58 heterozygous subjects. With this information we estimate the prevalence for heterozygosity at 1:1050 corresponding to an prevalence of homozygosity of 1:4,400,000 in this large pluriethnic cohort.

Diagnosing a mutation causing monogenic obesity will enable pharmacological treatment in some instances and is a psychological relief for the patient and the relatives. To facilitate its diagnosis, available information on variants in the leptin gene should be harmonized with associated phenotypic characteristics in a structured and comprehensive way by establishing an international registry for this rare disease.

## Additional file

Additional file 1: Table S1. List of genes in which mutations were described to affect the leptin-melanocortin signaling pathway [3, 6, 7, 9, 20, 36–46]. **Table S2.** Leptin gene variants described in the literature. Reference sequence for c.DNA: NM_000230/ Transcript ID: ENST00000308868.4 [3–19, 21, 22, 33, 47]. **Table S3.** Summary of missense and LoF variants in the leptin gene LEP listed in the ExAC database. Gray rows indicate mutations evaluated as probably functionally damaging by our own knowledge-driven functional assessment. (DOCX 69 kb)

## Abbreviations

BMI: Body mass index
BS: Binding Side
Del: Deletion
ExAC: Exome aggregation consortium
FAQ: Frequently asked questions
LEP: Leptin gene
LOFTEE: Loss-of-Function Transcript Effect Estimator
MC4R: Melanocortin 4 receptor
PolyPhen-2: Polymorphism phenotyping v2
SIFT: Sorting Intolerant from Tolerant
