# Supplementary material for: Estimated prevalence of potentially damaging variants in the leptin gene
Source: Mol Cell Pediatr. 2017 Nov 3;4:10. doi: 10.1186/s40348-017-0074-x (PMC5670095; doi:10.1186/s40348-017-0074-x)
Supplement: Additional file 1: Table S1. — List of genes in which mutations were described to affect the leptin-melanocortin signaling pathway [3, 6, 7, 9, 20, 36–46]. Table S2. Leptin gene variants described in the literature. Reference sequence for c.DNA: NM_000230/ Transcript ID: ENST00000308868.4 [3–19, 21, 22, 33, 47]. Table S3. Summary of missense and LoF variants in the leptin gene LEP listed in the ExAC database. Gray rows indicate mutations evaluated as probably functionally damaging by our own knowledge-driven functional assessment. (DOCX 69 kb) [file 40348_2017_74_MOESM1_ESM.docx]

**Table S 1: List of genes in which mutations were described to affect the leptin-melanocortin signaling pathway**

|  | Mutation and References | Way of action | Phenotype |
| --- | --- | --- | --- |
| Impaired leptin signaling | Leptin (LEP) e.g.: [3,6,7,9] | Secreted by adipocytes. Binds to the leptin receptor in the arcuate nucleus of the hypothalamus. Enhances expression of pro-opiomelanocortin (POMC) and melanocyte stimulating hormone (MSH) and inhibits antagonists of POMC action, namely neuropeptide Y (NPY) and agouti-related protein | -Normal birthweight - Severe early-onset obesity  -Intense Hyperphagia -Aggressive behavior when food is restricted -High energy intake on ad libitum test meals -Altered T-cell number and function (often higher infection rate) -Hypogonadotropic hypogonadism |
|  | Leptin-receptor (LEPR) e.g: [36,37,39] | Activated by leptin. Signal transmission e.g. via JAK2/STAT3, activating POMC and indirectly MC4R | See LEP |
|  | Scr homology 2B adapter protein 1 (SH2B1) e.g.: [38,40] | Important for downstream signaling in several receptor tyrosine kinases and cytokine receptors acting via JAK signaling e.g. receptors for leptin, GH, IGF-I and Insulin. Important role in enhancing JAK activation and controlling leptin sensitivity | -Leptin resistance -Severe early-onset obesity -High calorie intake -Insulin resistance -Delay in language development -Disturbed social behavior |
| Impaired melanocortin- signaling | Pro-opiomelanocortin (POMC) e.g.: [41,42] | Leptin signaling stimulates expression of POMC in primary neurons in the arcuate nucleus. POMC is a precursor protein for peptide hormones e.g. MSH, and adrenocorticotropin (ACTH) | -Hyperphagia  -Early-onset obesity  -ACTH related: hypoglycemia  -Caucasian subjects are often red-haired  -Pale skin |
|  | melanocortin-4 receptor (MC4R) e.g.: [20,43-46] | α-MSH is produced in the arcuate nucleus and binds to the MC4R receptor expressed in the paraventricular nucleus and the lateral hypothalamic area. Activation of MC4R by MSH increases expression of two anorexigenic peptides: corticotropin-releasing hormone (CRH) and thyrotropin-releasing hormone (TRH). MC4R decreases orexigenic actors: melanin-concentrating hormone (MCH) and orexin. | -Early-onset hyperphagia  -Increased lean mass  -Increased bone density  -Hyperinsulinemia  -Reduced symphaticus activity |

**Table S 2: Leptin gene variants with suggested functional impairment described in the literature.** Reference sequence for c.DNA: NM_000230/ Transcript ID: ENST00000308868.4

| Protein sequence immature^a^ leptin | Coding DNA sequence | Patients  (number) | References |
| --- | --- | --- | --- |
| p.Ile35*del* | c.104_106delTCA | 2^b^ | [13,14] |
| p.Gln55* | c.163C>T | 1 | [21] |
| p.Leu72Ser | c.215T>C | 1 | [6] |
| p.Asp100Tyr | c.298G>T | 1 | [7] |
| p.Asn103Lys | c.309C>A | 5 | [8,12,18] |
| p.Arg105Trp | c.313C>T | 7 | [4,5,17,22] |
| p.Cys117Tyr | c.350G>A | 1 | [17] |
| p.Trp121* | c.363G>A | 1 | [16] |
| p.Gly133Val*fs**15 | c.398delG | 30 | [3,9-11,13-15] |
| p.Ser141Cys | c.422C>G | 2 | [47] |
| p.Leu161Gly*fs**10 | *c.481_482delCT* | 1 | [13] |
| n.n | c.1-44del | 1 | [17] |
| p.His118Leu^c^ | c.353A>T | 1 | [19] |

^a^ leptin maturation includes the cleavage of a 21 amino acid long N-terminal signal peptide

^b^ possibly the same patient

*indicates a premature stop-codon

^c^This mutation in the leptin gene was published in 2014. As the described patient was heterozygous (not homozygous as the others) we excluded this variant from our counts.

**Table S 3: Summery of missense and LoF variants in the leptin gene LEP listed in the ExAC database.** Grey rows indicate mutations evaluated as probably functionally damaging by our own knowledge-driven functional assessment.

| Position | Consequence | Homozygous | Heterozygous | Allele frequency | PolyPhen-2 | SIFT |
| --- | --- | --- | --- | --- | --- | --- |
| 127892093 | p.Gly8Arg | 0 | 2 | 0.00001647 | Benign | Tolerated |
| 127892101 | p.Leu10Phe | 0 | 4 | 0.00003295 | Probably damaging | Tolerated |
| 127892105 | p.Leu12Ile | 0 | 1 | 0.000008236 | Possibly damaging | Tolerated |
| 127892115 | p.Tyr15Cys | 0 | 1 | 0.000008236 | Benign | Tolerated |
| 127892117 | p.Leu16Val | 0 | 1 | 0.000008236 | Possibly damaging | Tolerated |
| 127892124 | p.Tyr18Cys | 0 | 50 | 0.0004118 | Benign | Tolerated |
| 127892129 | p.Gln20Glu | 0 | 1 | 0.000008236 | Benign | Tolerated |
| 127892169 | p.Thr33Asn | 0 | 2 | 0.000008236 | Possibly damaging | Tolerated |
| 127892171 | p.Ile35del | 0 | 2 | 0.00001647 | In-frame deletion^a^ | |
| 127892178 | p.Lys36Arg | 0 | 2 | 0.00001647 | Benign | Tolerated |
| 127892204 | p.Ile45Val | 0 | 10 | 0.00008237 | Benign | Tolerated |
| 127892205 | p.Ile45Asn | 0 | 1 | 0.000008237 | Probably damaging | Deleterious |
| 127892211 | p.His47Gln*fs*Ter24 | 0 | 1 | 0.000008237 | LoF: High confidence^b^ (frameshift) | |
| 127892214 | p.Thr48Met | 0 | 2 | 0.00001647 | Benign | Tolerated |
| 127894455 | c.145-2A>G | 0 | 1 | 0.000008267 | LoF: High confidence ^b^ (consensus splice acceptor site) | |
| 127894467 | p.Ser52Phe | 0 | 1 | 0.000008259 | Probably damaging | Deleterious |
| 127894475 | p.Gln55Lys | 0 | 3 | 0.00002476 | Possibly damaging | Tolerated |
| 127894499 | p.Ile63Leu | 0 | 10 | 0.00008246 | Benign | Tolerated |
| 127894550 | p.Ala80Ser | 0 | 1 | 0.000008237 | Possibly damaging | Tolerated |
| 127894551 | p.Ala80Val | 0 | 1 | 0.000008237 | Benign | Tolerated |
| 127894568 | p.Leu86Phe | 0 | 1 | 0.000008237 | Possibly damaging | Tolerated |
| 127894580 | p.Pro90Thr | 0 | 1 | 0.000008237 | Benign | Deleterious |
| 127894592 | p.Val94Met | 52 | 909 | 0.007916 | Benign | Tolerated |
| 127894610 | p.Asp100Asn | 0 | 1 | 0.000008237 | Probably damaging | Deleterious |
| 127894621 | p.Asn103Lys | 0 | 1 | 0.00001647 | Probably damaging | Deleterious |
| 127894640 | p.Val110Met | 0 | 16 | 0.0001318 | Benign | Tolerated |
| 127894640 | p.Val110Leu | 0 | 1 | 0.000008238 | Benign | Tolerated |
| 127894679 | p.Ser123Gly | 0 | 2 | 0.00001653 | Benign | Tolerated |
| 127894692 | p.Thr127Ile | 0 | 1 | 0.000008291 | Possibly damaging | Deleterious |
| 127894704 | p.Gly133Val*fs*Ter15 | 0 | 4 | 0.00003322 | LoF: High confidence ^b^ (frameshift) | |
| 127894706 | p.Gly132Arg | 0 | 1 | 0.000008311 | Possibly damaging | Tolerated |
| 127894710 | p.Gly133Val | 0 | 1 | 0.000008317 | Benign | Tolerated |
| 127894721 | p.Ala137Ser | 0 | 1 | 0.000008340 | Benign | Tolerated |
| 127894767 | p.Gly152Glu | 0 | 1 | 0.000008478 | Possibly damaging | Tolerated |
| 127894778 | p.Asp156Asn | 0 | 1 | 0.00001701 | Benign | Tolerated |
| 127894792 | p.Gln160His | 0 | 2 | 0.00001720 | Probably damaging | Tolerated |
| 127894796 | p.Asp162Asn | 0 | 1 | 0.000008629 | Probably damaging | Deleterious |
| 127894800 | p.Leu163Arg | 0 | 2 | 0.00001731 | Benign | Tolerated |
| 127894802 | p.Ser164Arg | 0 | 1 | 0.000008655 | Possibly damaging | Tolerated |
| 127894808 | p.Gly166Arg | 0 | 2 | 0.00001734 | Benign | Tolerated |

^a^Not predicted by PolyPhen-2 or SIFT (these algorithms only predict the consequence of missense variants)

^b^Assessed with the help of LOFTEE
